# Nitrazepam and 7-aminonitrazepam studied at the macroscopic and microscopic electrified liquid-liquid interface

**DOI:** 10.1007/s00604-023-05739-6

**Published:** 2023-04-13

**Authors:** Paweł Stelmaszczyk, Karolina Kwaczyński, Konrad Rudnicki, Sławomira Skrzypek, Renata Wietecha-Posłuszny, Lukasz Poltorak

**Affiliations:** 1 Laboratory for Forensic Chemistry, Department of Analytical Chemistry, Faculty of Chemistry, Jagiellonian University, Gronostajowa 2, 30-387, Krakow, Poland; 2 Electrochemistry@Soft Interfaces Team, Department of Inorganic and Analytical Chemistry, Faculty of Chemistry, University of Lodz, Tamka 12, 91-403, Lodz, Poland

**Keywords:** Date rape drugs, Psychoactive chemicals, Voltammetry, ITIES, Electrochemical sensor

## Abstract

Two benzodiazepine type drugs, that is, nitrazepam and 7-aminonitrazepam, were studied at the electrified liquid-liquid interface (eLLI). Both drugs are illicit and act sedative in the human body and moreover are used as date rape drugs. Existence of the diazepine ring in the concerned chemicals structure and one additional amine group (for 7-aminonitrazepam) allows for the molecular charging below their pKa values, and hence, both drugs can cross the eLLI interface upon application of the appropriate value of the Galvani potential difference. Chosen molecules were studied at the macroscopic eLLI formed in the four electrode cell and microscopic eLLI formed within a microtip defined as the single pore having 25 μm in diameter. Microscopic eLLI was formed using only a few μL of the organic and the aqueous phase with the help of a 3D printed cell. Parameters such as limit of detection and voltammetric detection sensitivity are derived from the experimental data. Developed methodology was used to detect nitrazepam in pharmaceutical formulation and both drugs (nitrazepam and 7-aminonitrazepam) in spiked biological fluids (urine and blood).

**Graphical abstract:**

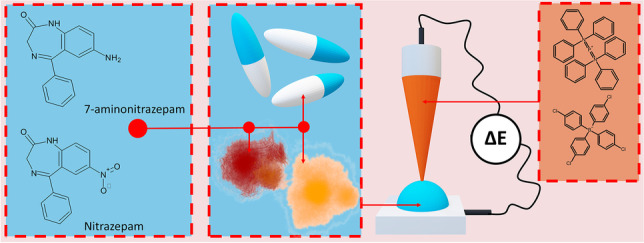

**Supplementary Information:**

The online version contains supplementary material available at 10.1007/s00604-023-05739-6.

## Introduction

Benzodiazepines (BZDs) were first used as alternative drugs to barbiturates with fewer side effects. They have a depressant effect on the central nervous system, acting anxiolytic, sedative, hypnotic, and anticonvulsant in the human body. Their action is based on the influence on the functioning of the GABA receptors system [1]. It is estimated that BZDs are one the most widely used group of drugs worldwide. Although their use has many positive effects, long-term intake is associated with the risk factors such as drowsiness, anterograde amnesia, sedation, and increased tolerance to drugs of this group [2, 3]. One of the most commonly available chemical from the BZDs family is nitrazepam, which, apart from its pharmacological use, is also used by offenders of drug-facilitated sexual assault (DFSA). This substance, compared to other substances from the benzodiazepine group, is characterized by a short onset time of 10–40 min, and its half-life in the human body of 18–38 h. The therapeutic dose of this drug is usually around 5–10 mg. The main metabolic products of this substance are two inactive metabolites: 7-aminoflunitrazepam and 7-acetamidonitrazepam [4]. In the case of therapeutic drug monitoring in pharmacological treatment, as well as in the case of DFSA crimes, it is necessary to use methods that enable the detection of nitrazepam and its metabolites in various types of samples (tablets and biological material such as blood and urine). In routine analyzes of these substances, chromatographic [5–8] and spectrophotometric methods [9, 10] are usually found. Electrochemical sensors provide an alternative that requires little amount of chemicals very low operational price of the needed hardware, and often enable the analysis of the sample without any preliminary preparation.

Electrochemical sensors allowing for a rapid determination of the illicit drugs attracts significant attention as a presumptive replacement for the colorimetric tests [11–14]. The focus of the recent development is especially given to street formulation (street samples–illicit drugs enriched by the cutting agents). Elegant and fully functional examples of simple electrochemical procedures to detect chosen illicit drugs in the presence of common adulterants are frequently based on simple solutions. A series of works focused on cocaine detection simply derived from the oxidation of its tertiary amine at carbon based electrodes (either bare or modified with conductive nanomaterials) reveled a voltammetric profile of the concerned drug, and its frequently used cutting agents [15–17]. Still, emerging effort is given to the electrochemical detection of other popular drugs such as heroin [18–20], amphetamine [21–23], fenantyl [24], morphine [25], or tetrahydrocannabinol [26, 27] being significantly less popular as compared with cocaine [14]. Most of the reports available in the literature which are focused on the development of the electrochemical sensors for the detection of illicit drugs are based on the carbon-based electrodes, direct approach (ideal material for amine functionalities oxidation), or conductive material modified with a range of different (nano/bio)objects influencing sensors electroanalytical parameters (detection limits, sensitivity and selectivity) [14, 28–30]. For the latter, the example of common modifiers include conductive polymers [31], carbon nanoobjects [32],  aptamers [33–36] (predominantly for cocaine), and multilayered configurations [37].

The promising sensing system that allows for the selective detection of different classes of illicit drugs, also being employed in this work, is based on the electrified liquid-liquid interface. In the text we refer to it as a well-recognized abbreviation, ITIES, meaning the interface between two immiscible electrolyte solution [38–43]. Instead of a solid electrode used to follow redox reaction, ITIES profits from the signal in a form of ion transfer being recoded at the soft junction formed between two mutually immiscible solutions [44–47]. Even though interfacial electron transfer reaction driven by a Galvani potential difference is possible, from an electroanalytical point of view, the utility of this type of an interfacial charge transfer type is still limited to a nanoobjects detection [48, 49]. Detection at ITIES is governed by the molecular partitioning properties of the studied compounds. This feature is highly useful when developing a sensing platform that aims to detect compounds from a mixture of a chemical species holding a shared core chemical structure with different substituents and displaying different partitioning properties [50–54]. Downscaling of the contact between immiscible phase (miniaturization) has many beneficial consequences. With the lower surface area, we diminish the contribution of the capacitive currents and alter the shape of the diffusion layer profiles which improve the electroanalytical properties of the sensing systems [55–58]. ITIES miniaturization requires an appropriate support meeting a few prerequires: (i) asymmetric properties (sharp boundary between hydrophobic and hydrophilic domains) of the support wettability should assure the stability of the ITIES position; (ii) it must provide the apertures with micrometer (or less) dimensions that can be filled with either phase (aqueous or organic) prior to contacting with the immiscible solution; and (iii) the apertures height should not significantly exceed the ITIES dimeter as it will become a reason of the additional resistance. In practice, the employed supports have a form of fabricated chips with precisely arranged nano/micrometer pores [45, 50, 59–61], inherently porous membranes [62–64], or capillaries with a defined pore diameter [18, 65–70].

In this work, we have studied the interfacial behavior of nitrazepam (NIT) and 7-aminonitrazepam (7a-NIT). Both drugs are a class of benzodiazepines being depressant drugs which are frequently misused as the date rape drugs. Our work aims at understanding the interfacial behavior of both molecules. We provide a number of physicochemical, electroanalytical, and pharmacochemical parameters that are directly extracted from the voltammetric results obtained at the macroscopic liquid-liquid interface. Next, we have used the miniaturization protocol which is based on the fused silica capillary and profred the electroanalytical study aiming at detecting NIT and 7a-NIT in pharmaceutical formulation, spiked urine and serum.

## Methods and materials

### Chemicals

Chemical used for the aqueous phase preparation: water demineralized using hydrolab water purification system, sodium chloride (NaCl, analytical grade, POCh), chloric acid (HCl, 35–38%, for analysis, ChemPure), sodium hydroxide (NaOH, for analysis, POCh), boric acid (H_3_BO_3_, for analysis, POCh), acetic acid (CH_3_COOH, 99.8%, for analysis, ChemPure), phosphoric acid (H_3_PO_4_, 80%, for analysis, ChemPure), tetramethylammonium chloride (TMACl, > 98%, Acros Organics), tetrapropylammonium chloride (TPrACl, > 99%, Alfa Aesar), 7-aminonitrazepam (7a-NIT, 1 mg·mL^−1^ in acetonitrile, > 98.5%, Tusnovics), and nitrazepam (NIT, 1 mg·mL^−1^ in acetonitrile, > 98.5%, Tusnovics).

Chemicals used for the organic phase preparation: 1,2-dichloroethane (1,2-DCE, ReagentPlus, 99%, Sigma), potassium tetrakis(4-chlorophenyl)borate (KTPBCl, > 98%, Merck), and bis(triphenylphosphoranylidene)ammonium chloride (BTPPACl, 97%, Merck).

Britton Robinson Buffer: 10 mM NaCl, 10 mM H_3_BO_3_, 10 mM H_3_PO_4_ and 10 mM CH_3_COOH solution with the pH adjusted using 1 M NaOH.

Organic phase electrolyte: BTTPATPBCl was precipitated and purified from a mixture of 0.1 g·mL^−1^ BTPPACl and 0.1 g·mL^−1^ KTPBCl each dissolved in 1:1 (volume) MeOH:H_2_O.

During all experiments, all measurements were performed based on the dissolution of certified reference materials of nitrazepam and 7-aminonitrazepam for which total uncertainties were 0.8% and 1.0% of nominal value (1 mg mL^−1^), respectively. The calibration curves, the standard addition method in case of determination nitrazepam in pill, and spiking biological samples were prepared using these certified reference materials.

### Electrochemical measurements

All electrochemical measurements were performed using Autolab 302n with either 4-electrode or 2-electrode configuration. Traditional four electrode electrochemical glass cell with two platinum electrodes serving as the counter electrodes and two Ag/AgCl wires—reference electrodes—immersed into Luggin capillaries (liquid-liquid interface was always positioned in between two capillaries) is referred to as macroITIES. Experiments performed in a microscopic cell (microITIES) are based on a 3D printed platform used as the support for the aqueous phase (experiments in a droplet having a volume of 5–10 μL) [18]. Here, the LLI interface was supported with the fused silica capillary tubing hosted in the plastic casing; fabrication details are reported elsewhere [71].

#### 3D printing

The 3D printed platforms used as the reservoirs for the aqueous phase droplet and support for the fused silica microcapillaries were printed using fused deposition modeling (FDM) technology with the Prusa i3 MK3S+ printer. All designs were made in the Thinkercad and then exported as the STL files. Printout was made with the polylactic acid (PLA) based filament by Fiberology. Prusa slicer was used to set the G-code. The printing parameters are as follows: print-out quality, 0.15 mm; filament type, Generic PLA; infill, 60%; nozzle temperature for the first layer, 215 °C; nozzle temperature for other layers, 210 °C; and bed temperature for all layers, 60 °C. Silver wire covered with Ag/AgCl layer was placed in the cell middle compartment and was further secured with the silicone sealant.

### Microwave-assisted extraction

The microwave-assisted extraction (MAE) protocol was performed based on report published by Stelmaszczyk et al. [5] In brief, 500 μL of whole blood or urine spiked with NIT or 7a-NIT was pipetted into Teflon vessels, and next extraction mixture was added (1 mL of buffer pH = 9 borax/hydrochloric acid and 3 mL of ethyl acetate). The MAE extraction was carried out at 50 °C for 15 min. After extraction, the contents of the vessels were transferred to the 15-mL plastic tubes and centrifuged (4000 rpm, 4 °C, 5 min). Next, 2.5 mL of extractant was taken and evaporated under nitrogen gas at a temperature of 40 °C. To the residue, 500 μL of ethyl acetate was added. Then, the sample was vortexed for 10 s, and next centrifuged (10,000 rpm, 4 °C, 10 min). The portion of 450 μL was taken and again was dried under nitrogen gas at 40 °C. Before the electrochemical measurements, the sample was reconstituted in 50 μL of 10 mM HCl and 10 mM NaCl used as the aqueous phase in the eLLI electrochemical configuration. The biological samples were always spiked with tested analytes before an analytical procedure was applied for the samples treatment.

### Protocol for unknown pill sample solution preparation

The pill subjected to investigation was crashed and dissolved in 25 mL of 10 mM HCl and 10 mM NaCl solution. Next, the dissolution process was supported by ultrasonication for 30 min, after which the solution was filtered using PTFE filters (0.45 μm). In next step, filtered solution was diluted in 10 mM HCl and 10 mM NaCl solution (20 μL of sample and 980 μL of aqueous phase). Afterwards, in order to prepare the standard addition calibration curve, the signals were recorded for samples without and with standard additions. First, the 10 μL of diluted sample was placed in 3D printed cell, and the voltammetric curve was registered. Next, the 4 measurements were performed with the following 4 additions of 80 μM analyte standard solution in aqueous phase; each addition of the standard was 2.5 μL. The signal at around 0.240 mV was analyzed, and then a calibration curve was plotted by the standard addition series method. Next, by extrapolating the curve to the point of intersection with the *x* axis and taking into account the dilution steps of the sample, the content of the analyte in the pill was determined.

## Results and discussion

**Scheme 1:**
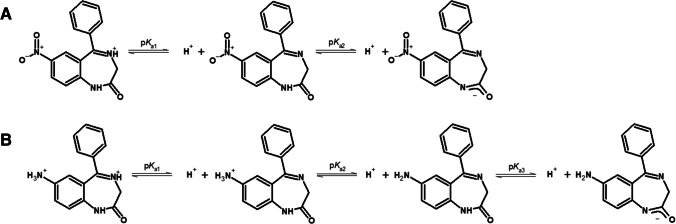
Acid-base equilibria for nitrazepam (**A**; NIT; p*K*_a1_ = 3.2; p*K*_a2_ = 10.8)[72] and 7-aminonitrazepam (**B**; 7a-NIT; p*K*_a1_ = 2.5; p*K*_a2_ = 4.6; p*K*_a3_ = 13.1) [73]

The common chemical feature of all benzodiazepine drugs is the presence of substituted benzene and diazepine rings (benzodiazepine units). From the ITIES point of view, the heterocycle and eventually ionizable substitutes (e.g., primary amine group in the 7a-NIT) under proper conditions defined by the acid base equilibria assures the presence of the charge located within the molecule structure allowing for its electrochemically controlled interfacial ion transfer. Different species of NIT and 7a-NIT are shown in Schemes 1A and B, respectively. The only difference between the studied analytes is the benzene ring substituent being nitro group for the NIT and primary amine group for the 7a-NIT. First, p*K*_a_ value for nitrazepam was reported to be 3.2 and corresponds to the protonation of the nitrogen atom located at the position 4 in the benzodiazepine ring [72]. Protonation of the analogical position in the 7a-NIT structure is defined by the p*K*_a_ = 2.5 [73]. For the latter, further increase in pH is associated with the protonation of the primary amine group substituted to the benzene ring (p*K*_a_ = 4.6). For both molecules, the highest p*K*_a_ values, that is, 10.6 for NIT and 13.1 for 7a-NIT, correspond to the protonation/deprotonation of the azomethine group [74].  As such, within the conventional pH range, we can distinguished three species of NIT (monocationic at pH < 3.2, neutral at 3.2 < pH < 10.8, and monoanionic at pH > 10.8) and four species of 7a-NIT (dicationic at pH < 2.5, monocationic at 2.5 < pH < 4.8, monoanionic at pH > 13.1, and neutral at 4.8 < pH < 13.1).

**Fig. 1:**
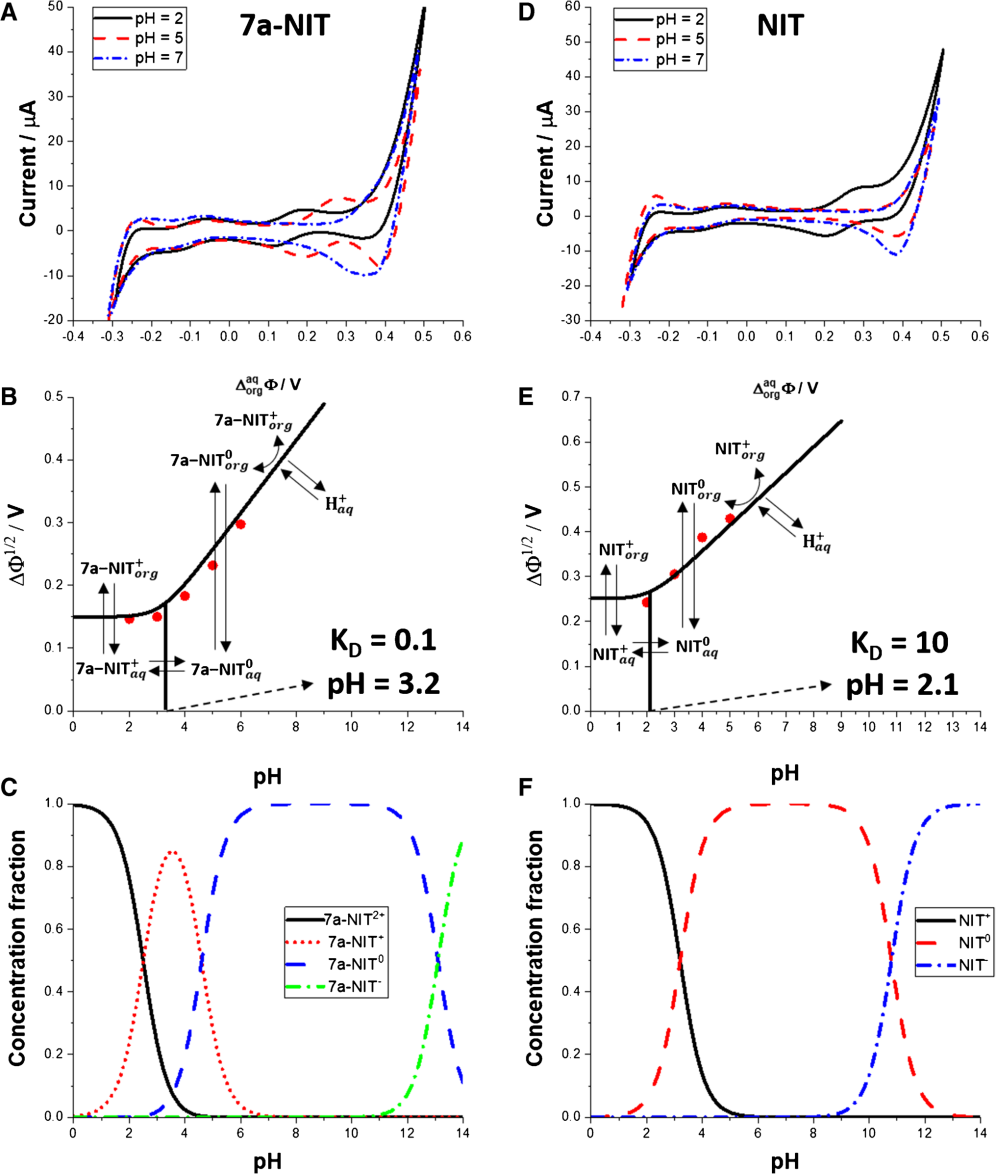
Ion transfer voltammograms (ITVs) recorded for 7a-NIT (**A**) and NIT (**D**) at pH equal to 2.0 (black, solid line); pH = 5.0 (red, dashed line) and pH = 7.0 (blue, dash-dot line). Scan rate was 20 mV·s^−1^. Signals appearing at − 160 mV originate from the simple ion transfer of the [TPrA^+^] = 30 μM used as the internal reference probe. Ion partitions diagrams for 7a-NIT and NIT are given as part (**B**) and (**E**), respectively. For details, refer to the main text. (**C**) (7a-NIT) and (**F**) (NIT) are the concentration fraction diagrams. For p*K*_a_ values, refer to Scheme 1

**Fig. 2:**
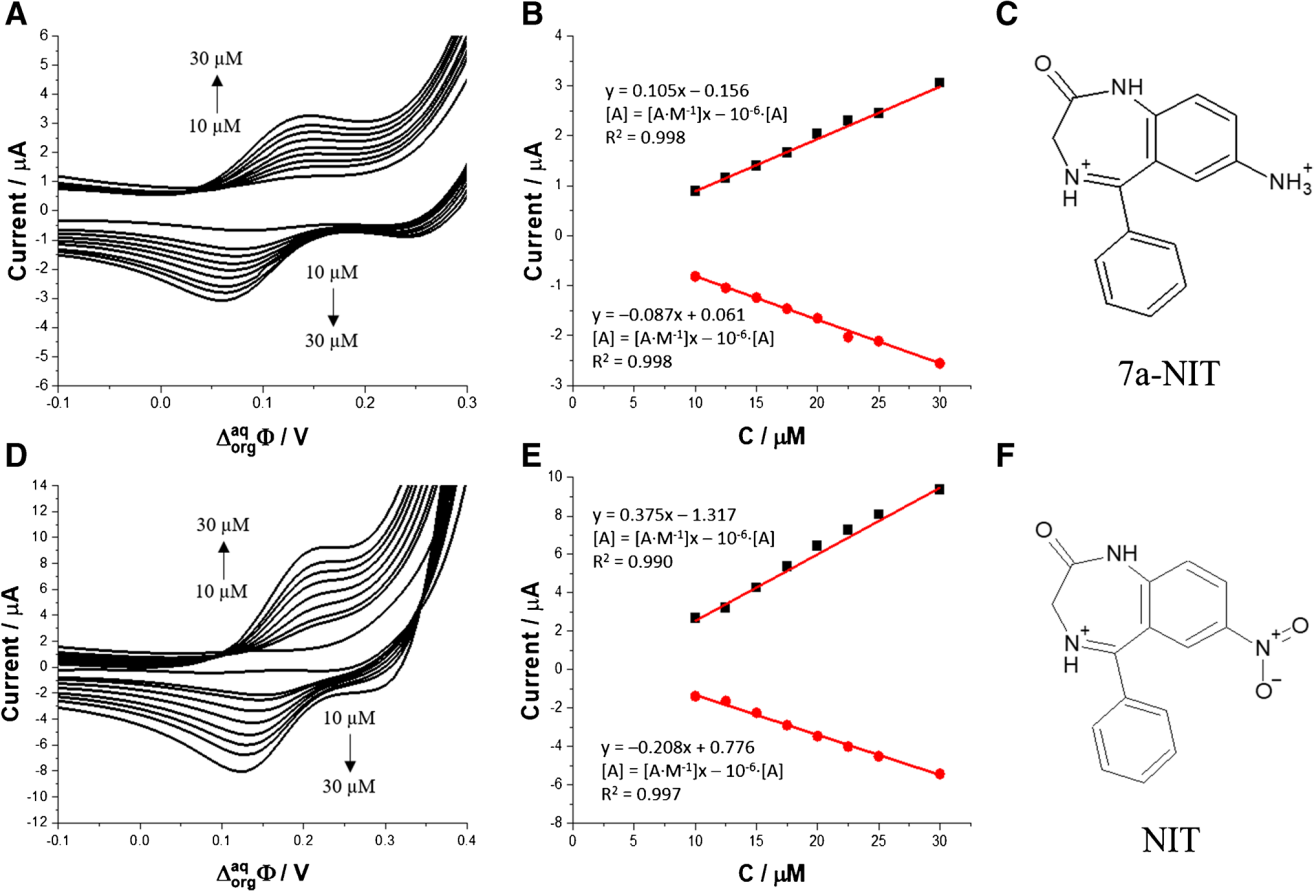
(**A**) and (**D**) represent ion transfer voltammograms (scan rate = 20 mV·s^−1^) recorded for increasing concentration (10.0; 12.5; 15.0; 17.5; 20.0; 22.5; 25.0; 30.0 μM) of 7a-NIT and NIT, respectively. The aqueous phase: 10 mM NaCl, 10 mM HCl, and pH = 2.0. (**B**) and (**E**) are the calibration curves plotted based on the forward (positive) and backward (negative) peaks’ current intensities recorded for 7a-NIT and NIT, respectively. (**C**) and (**F**) show the chemical structures of 7a-NIT and NIT, respectively

Fig. 1 summarizes ITVs recorded at pH 2.0; 5.0 and 7.0 (additional voltammograms recorded in the pH range from 2.0 to 9.0 are available in electronic supporting information as Fig. S1,; ion partition diagrams, and concentration fraction diagrams. We have found that the signal originating from the 7a-NIT could be clearly recorded starting from pH 2.0 and up to pH = 6.0 (Fig. 2A and Fig. S2A), whereas for NIT, a pair of two peaks recorded within the more positive Galvani potential difference scale could be recorded only for pH range from 2.0 until 5.0 (Fig. 2B and Fig. S2B). ${\Delta }_{org}^{aq}{\phi}_{benzodiazepine}^{\prime }$ was extracted from voltammetric curves for each drug dissolved in the aqueous phase having different pH and plotted in a form of ion partition diagram (see Fig. 2C for 7a-NIT and Fig. 2D for NIT). Red circles correspond to the experimental data, whereas solid black lines represent the boundary lines that were plotted using the following equation:

1 $${\Delta }_{org}^{aq}{\phi}_{benzodiazepine}={\Delta }_{org}^{aq}{\phi}_{benzodiazepine}^{\prime }+\frac{RT}{F}\left(\frac{10^{- pH}+{K}_a+{K}_a{K}_D}{10^{- pH}}\right)$$

where ${\Delta }_{org}^{aq}{\phi}_{benzodiazepine}^{\prime }$ is the formal Galvani potential of the protonated benzodiazepine drug (either 7a-NIT^+^ or NIT^+^), *K*_a_ is defined by the p*K*_a_ (acid dissociation constant) of the 7a-NIT (p*K*_a2_) or NIT (p*K*_a1_), whereas *K*_D_ is the distribution constant defined as the concentration ratio of the neutral form of the drug dissolved in the aqueous and the neutral form of the drug dissolved in the organic phase. *K*_D_ is the variable in Eq. 1 allowing for the adjustment of the fitted data. *K*_D_ for 7a-NIT was found to be equal to around 0.1, whereas for NIT, is was estimated to be around 10. This means that 7a-NIT in its neutral form is significantly more hydrophilic as compared to neutral form of NIT. The pH dependent mechanism of the interfacial charge transfer reaction can be directly deducted from the ion partition diagrams. For 7a-NIT, based on the obtained data, we have concluded that the ionic currents at ${\Delta }_{org}^{aq}\phi$ from 100 to 500 mV and up to pH = 4.0 are governed by the simple ion transfer of the monoprotonated 7a-NIT^+^ rather than doubly protonated 7a-NIT^2+^ whose concertation fraction in the aqueous phase at pH = 2.0 was calculated to be 0.75. Since 7a-NIT^2+^ is less lipophilic than 7a-NIT^+^, its transfer should occur at more positive ${\Delta }_{org}^{aq}\phi$ that in our case will be overlaid with the interfacial transfer of H^+^ (pH = 2.0; ${\Delta }_{org}^{aq}{\phi}_{H^{+}}^{\prime }$ = 549 mV) [75] or even will occur at the more positive potential values falling beyond available potential window. Also, we have observed that the positive peak current attributed to the 7a-NIT^+^ transfer from the aqueous to the organic phase increased from 19 to 23 μA (17%) when changing the pH from 3.0 to 4.0. From Fig. 1C, one can deduce that the concentration fraction of 7a-NIT^+^ increased from 71% to 82%. This is in line with what was observed by others for dibasic species such as quinine [76, 77], nicotine, or hydralazine [78]. As such, we have concluded that the ionic signals recorded at 100 mV < ${\Delta }_{org}^{aq}\phi$ < 500 mV and in the pH range from 2.0 to 4.0 are due to a simple 7a-NIT^+^ interfacial ion transfer reaction. In other words, at ${\Delta }_{org}^{aq}\phi$ > 150 mV 7a-NIT^+^ partitions to the organic phase which requires applied Galvani potential difference, whereas at ${\Delta }_{org}^{aq}\phi$ < 150 mV, 7a-NIT^+^ will exists only in the aqueous phase. At pH > 4.0, the fraction of the 7a-NIT^0^ in the aqueous phase increases significantly and reaches unity at pH = 6.0 (see Fig. 1C). In this pH range, the recorded ionic currents are due to the facilitated transfer of proton from the aqueous phase by the neutral 7a-NIT^0^ species partitioned to the organic phase. According to Fig. 1F, the concentration fraction of NIT^+^ drops to 0 at pH = 5.0. Below this value, NIT exists in the aqueous phase as the monocationic specie reaching concentration fraction equal to unity at pH < 1. Similarly to 7a-NIT^+^, the signals recorded at 200 mV < ${\Delta }_{org}^{aq}\phi$ < 500 mV and up to pH = 4.0 are due to the simple NIT^+^ interfacial ion transfer reaction. Above pH = 4.0 we are expecting to observe the facilitated transfer of proton by the NIT^0^ that spontaneously partition to the 1,2-DCE phase. All these indicate that both studied molecules may undergo ion transfer reaction across the eLL, and, hence, can be detected with available electroanalytical tools.

Since both drugs studied in this work, this is 7a-NIT and NIT, were found to be electrochemically active, we have performed the electroanalytical study. Figure 2A and D are the ion ITVs recorded in the presence of the increasing concertation of 7a-NIT and NIT, respectively, initially added to the aqueous phase. Both benzodiazepines transfer falls for the relatively positive range of the formal Galvani potential difference (${\Delta }_{org}^{aq}{\phi}^{\prime }$) indicating their high hydrophilicity. The ${\Delta }_{org}^{aq}{\phi}_{7a-{NIT}^{+}}^{\prime }$ was found to be equal to 150 mV, whereas for ${\Delta }_{org}^{aq}{\phi}_{NIT^{+}}^{\prime },$ it is 242 mV. The ${\Delta }_{org}^{aq}{\phi}^{\prime }$ can be directly used to calculate the water-1,2-DCE portion coefficient (${logP}_{water/1,2- DCE}^{\prime }$):

2 $${logP}_{water/1,2- DCE}^{\prime }=-\frac{\Delta _{org}^{aq}{\phi}_{drug}^{\prime }{z}_iF}{2.303 RT}$$

where *z*_i_ is the charge of the molecule at given pH (z = 1) and *F*, *R*, and *T* have their usual meaning. Assuming Eq. 2, we have calculated and tabulated (see Table 1) partition coefficient values for 7a-NIT and NIT that are equal to – 2.58 and – 4.16, respectively. This clearly shows that monocationic NIT with the charge located at the nitrogen atom of the diazepine ring (position 4) is more hydrophilic then 7a-NIT with the charge located at the primary amine group substituent. As the aqueous phase concentration of the analytes reached 10 μM and was further elevated, we started observing signals with increasing current intensities that were attributed to the transfer of protonated and, hence, positively charged benzodiazepines from the aqueous to the organic phase (positive current) and from the organic to the aqueous phase (negative current). Obtained calibration curves are shown in Fig. 2B and E for 7a-NIT and NIT, respectively. We have found that the voltammetric detection sensitivity for the 7a-NIT (0.105 A·M^−1^ for positive current signals and 0.087 A·M^−1^ for negative current signals) holds the same order of magnitude as compared with the detection sensitivity obtained for NIT (0.375 A·M^−1^ for positive current signals and 0.208 A·M^−1^ for negative current signals). Figure S2 shows the ITVs recorded for the fixed concentration of 7a-NIT (Fig. S1A) and NIT (Fig. S1B). From the slope of the recorded current plotted in function of the square root of the applied potential scan rate that was substituted to a re-arranged Randles-Sevcik equation [75], we have calculated diffusion coefficient for both monoprotonated compounds having a concentration calculated using the concertation fraction diagrams (see Fig. 1C and F). Obtained values are equal to 11.2 and 14.1 ·10^−6^ cm^2^·s^−1^ for 7a-NIT^+^ and NIT^+^, respectively, which indicates that both monocationinc species hold similar hydrodynamic radii. Since the charge of the analyte and its diffusion coefficient directly affect the electroanalytical performance of the system [79], it is not surprising that the detection sensitivity for NIT is higher than its structural analogue 7a-NIT.

**Table 1:** Analytical parameters of 7a-NIT and NIT obtained at the macro- and μITIES systems. (+) – positive signals defined as the cation transfer from the aqueous to the organic phase; (–) – negative signals defined as the cation transfer from the organic to the aqueous phase

Parameter	macroITIES		μITIES	
	7a-NIT	NIT	7a-NIT	NIT
Studied LCR (μM)	10–30	10–30	2–30	2–30
Sensitivity				
(A·M^−1^) for macroITIES (nA·μM^-1^) for μITIES	0.105 (+) − 0.087 (–)	0.375 (+) − 0.208 (–)	0.38·10^−2^ (+) − 0.69·10^−2^ (–)	0.44·10^−2^ (+) − 0.32·10^−2^ (–)
Sensitivity*				
(A·M^−1^·cm^−2^) for macroITIES (A·M^−1^·cm^−2^) for μITIES	0.079 (+) − 0.066 (–)	0.283 (+) − 0.157 (–)	7745.22 (+) − 14063.69 (–)	8968.15 (+) − 6522.29 (–)
Intercept				
(μA) for macroITIES (nA) for μITIES	0.156 (+) 0.061 (–)	1.317 (+) 0.776 (–)	-	-
Coefficient of determination (*R*^2^)	0.998 (+) 0.998 (–)	0.990 (+) 0.997 (–)	0.987 (+) 0.996 (–)	0.975 (+) 0.980 (–)
LOD (μM)	0.71 (+) 1.23 (–)	2.15 (+) 1.86 (–)	0.21 (+) 0.42 (–)	0.42 (+) 0.38 (–)
${{\Delta }}_{{org}}^{{aq}}{{\phi}}^{\prime }$ (mV)	0.150	0.242	-	-
logP_1,2-DCE_	– 2.58	– 4.16	-	-
D (cm^2^·s^−1^)	11.2·10^−6^ (aq) 11.1·10^−6^ (org)	14.1·10^−6^ (aq) 13.1·10^−6^ (org)	-	-

^*^The electroactive surface area taken for the macroITIES and microITIES were calculated based on the radius equal to 0.65 cm and 12.5**·**10^-6^ cm, respectively. In both cases, it is assumed that ITIES takes the shape of a circle

**Figure 3:**
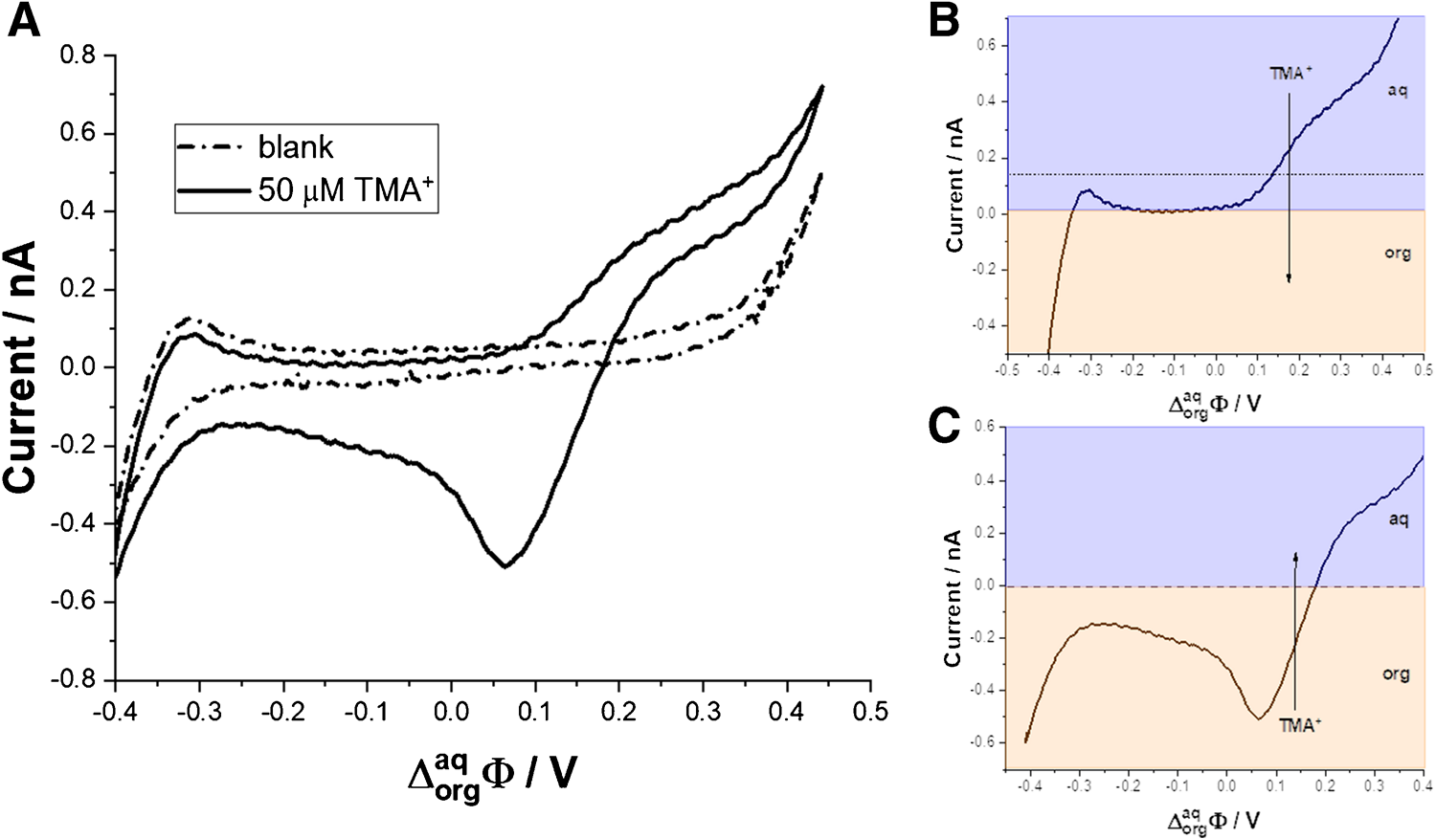
**A** Ion transfer voltammogram recorded at the ITIES supported with fused silica capillary in the absence (dash-dot line) and in the presence of the 50 μM TMA^+^. **B** The forward (positive) current for the transfer of TMA^+^ ions from the aqueous to the organic phase. **C** The backward (negative) current recorded for the TMA^+^ transfer from the organic to the aqueous phase. Conditions: scan rate = 20 mV·s^−1^; [TMA^+^] = 50 μM. SEM image of the fused silica capillary can be found in ESI

Electroanalytical studies were also performed at the μITIES formed within a single micropore defined by the dimeter of the fused silica microcapillary (25 μm) with the commercially preadjusted hydrophobicity of the pore interior (methyl deactivated surface). Capillary was supported with the polymeric casing and fabricated as described elsewhere [71]. The utilization of self-fabricated μITIES platforms was always proceeded by the voltammetric quality control step. Figure 3A shows the ITV recorded at μITIES in the absence and in the presence of [TMA^+^] = 50 μM. Addition of the model ion electrochemically active at the eLLI gives two signals. (i) Positive current having a shape of a semi-sigmoidal wave (see Fig. 3B for the first half-cycle of the ITV) which is due to the transfer of the TMA^+^ from the aqueous phase to the LLI located at the pore ingress and further to the organic phase. The shape of the recorded positive signal is not surprising as it is expected for the diffusion non-limited mass transfer governed by the hemispherical diffusion layer profile [60, 80–82]. The back transfer (from more to less positive Galvani potential difference, see Fig. 3C) forms a clear peak. The sign of the signal indicates that the TMA^+^ is back transferring from the organic to the aqueous phase. The shape of the signal clearly indicates that within the pore channel; the mass transfer is governed by the linear diffusion, and hence, peak instead of a wave is recorded [81, 83–85]. The electroanalytical μITIES support fabrication quality control is based on monitoring semi-sigmoidal (positive) signal intensity based on which the diameter of the formed ITIES is calculated using rearranged Saito equation (Eq. 3) [86, 87]:

3 $$r=\frac{I_{ss}}{4{z}_i DCF}$$

where *r* is the ITIES radius which is considered equivalent to the fused silica capillary pore radius; *I*_ss_ is the semi-steady state current (positive signal intensity) taken from the ITV; for quaternary ammonium cations (in this case TMA^+^), *z* is charge equal to 1, *D* is the diffusion coefficient (for TMA^+^ = 13.8 ·10^−6^ cm^2^·s^−1^), *C* is the concentration of the quaternary ammonium cation ([TMA^+^] = 50 μM), whereas *F* is the Faraday constant. According to Eq. 3, the μITIES support used to record ITV from Fig. 3A had a dimeter equal to 24 μm. The diameter of all capillaries employed in this study was always found in the range from 23.9 to 24.7 μm being slightly lower than the diameter claimed by the manufacturer (25 μm) but fully in line with what was observed using scanning electron microscopy [71].

**Fig. 4:**
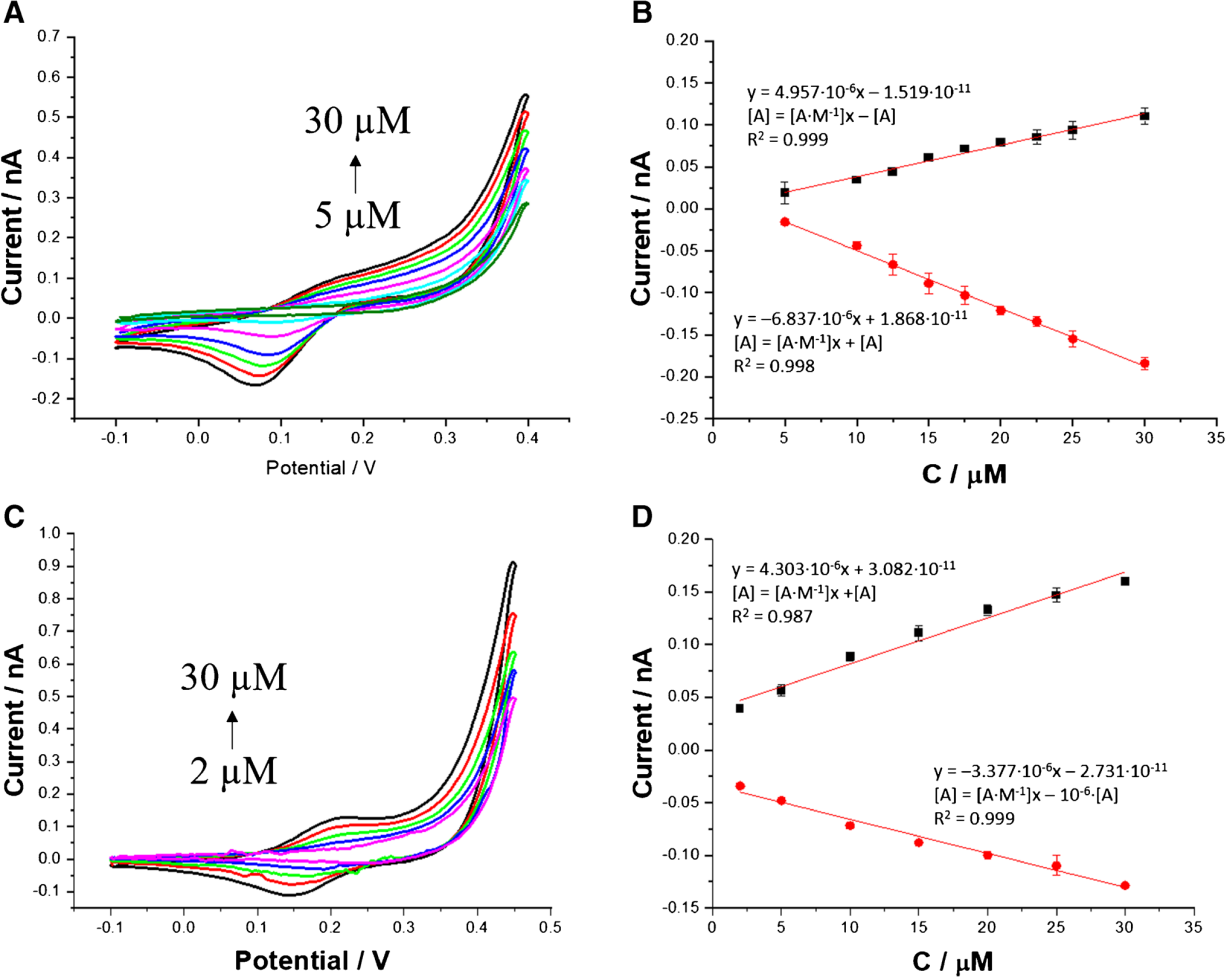
Selected ITVs recorded for increasing 7a-NIT (**A** 5.0; 10.0; 12.5; 15.0; 17.5; 20.0; 22.5; 25.0 and 30 μM) and NIT (**C** 2.0; 5.0; 10.0; 15.0; 20.0; 25.0; 30.0) at pH = 2. Calibration curves show the intensities of the forward (positive) and backward (negative) currents plotted in function of the [7a-NIT] (**B**) and [NIT] (**D**). Conditions: scan rate = 20 mV·s^−1^. Cyclic voltammograms from parts A and C were subjected to smoothing using adjacent averaging method (number of points = 5). In parts B and D, the error bars are present, although in some cases may be overlaid with the data points

In a next step, μITIES platforms were employed to develop electroanalytical procedure for the detection of 7a-NIT and NIT utilizing small amounts of the aqueous and the organic phases. Figure 4 shows the ITVs recorded in the presence of increasing concertation of 7a-NIT (Fig. 4A), and NIT (Fig. 4C) initially presents in the aqueous phase. The first concentration for which we were able to detect a measurable signal was equal to 5 μM and 2 μM for 7a-NIT and NIT, respectively.

Obtained values are two and five times higher as compared with the macroscopic system. This is not surprising as one of the advantages of the miniaturized platforms is lower value of the capacitive currents (electroactive surface area decreased by a few orders of magnitude) directly resulting in the decreasing limits of detections. The calibration curves for 7a-NIT and NIT are given along with the linear fit parameters in Fig. 4B and Fig. 4D, respectively. Obtained voltammetric detection sensitivities (e.g., 4.96·10^−6^ A·M^−1^ for 7a-NIT or 4.30·10^−6^ A·M^−1^ for NIT) hold expected order of magnitude and are similar to the values reported in literature [87–89]. From calibration curves linear fit equations, we have extracted fitting parameters that were further used to calculate limit of detection (LOD). Values of LOD were calculated based on the 3σ/a criteria, where σ was taken as the standard deviation of the intercept and a as the slope of the calibration curve. For example, LOD for 7a-NIT studied using μITIES was calculated to be 0.21 μM and was around 3 times lower than LOD for the same analyte studied at the macroscopic ITIES. LOD, LOQ, and voltammetry detection sensitives for both platforms (macro- and microscopic) and two studied analytes are compared in Table 1. The *R*^2^ values were found in the range from 0.975 to 0.998. Lowered values of the coefficient of determination may originate from the interfacial transient adsorption process, which can occur for molecules having the dimensionality (hydrodynamic radius) similar to benzodiazepines studied in this work [43].

To further confirm the high utility of the fabricated platforms based on the fused silica microcapillaries for the chosen benzodiazepines detection, we have calculated the relative standard deviation (RSD). The RSD errors were calculated based on the signals registered for 3 samples of NIT at the same concentration using 3 different 3D fabricated platforms. The values were calculated according to two approaches: (i) RSD calculated for 3 samples for each tested platform 1–3 (Table 2), and (ii) RSD calculated for 3 platforms for each tested sample 1–3 (Table 3). Results indicate that both forward and backward signals registered for 3 independent samples on one platform are repeatable (Table 2, with RSD < 1% and < 2%, respectively). Only in one case, for the backward signal calculated for platform 1, we have obtained slightly higher error, RSD = 6.9%, still being within the acceptable range. RSD values calculated for signals registered for each sample at 3 different platforms were below 5% which suggests that obtained results are repeatable when using different 3D printed platforms (Table 3).

**Table 2:** RSD calculated for three different platforms based on the samples 1, 2, and 3

Repetition	Samples 1–3				
	Added concentration/μM	Found concentration/μM	Recovery/% (*n* = 3)	Forward (positive) current, RSD/% (*n *= 3)	Backward (negative) current, RSD/% (*n* = 3)
Platform 1	30.0	31.0	103.3	0.1	6.9
Platform 2		29.1	96.9	0.9	0.1
Platform 3		30.1	100.4	0.7	1.9

**Table 3:** RSD calculated for three different samples each studied on the platforms 1, 2, and 3

Sample	Platforms 1–3				
	Added concentration/μM	Found concentration/μM	Recovery/% (*n* = 3)	Forward (positive) current, RSD/% (*n* = 3)	Backward (negative) current, RSD/% (n = 3)
Sample 1	30.0	30.1	100.5	3.1	4.3
Sample 2		29.9	99.8	4.1	4.1
Sample 3		30.6	102.1	4.9	4.6

**Fig. 5:**
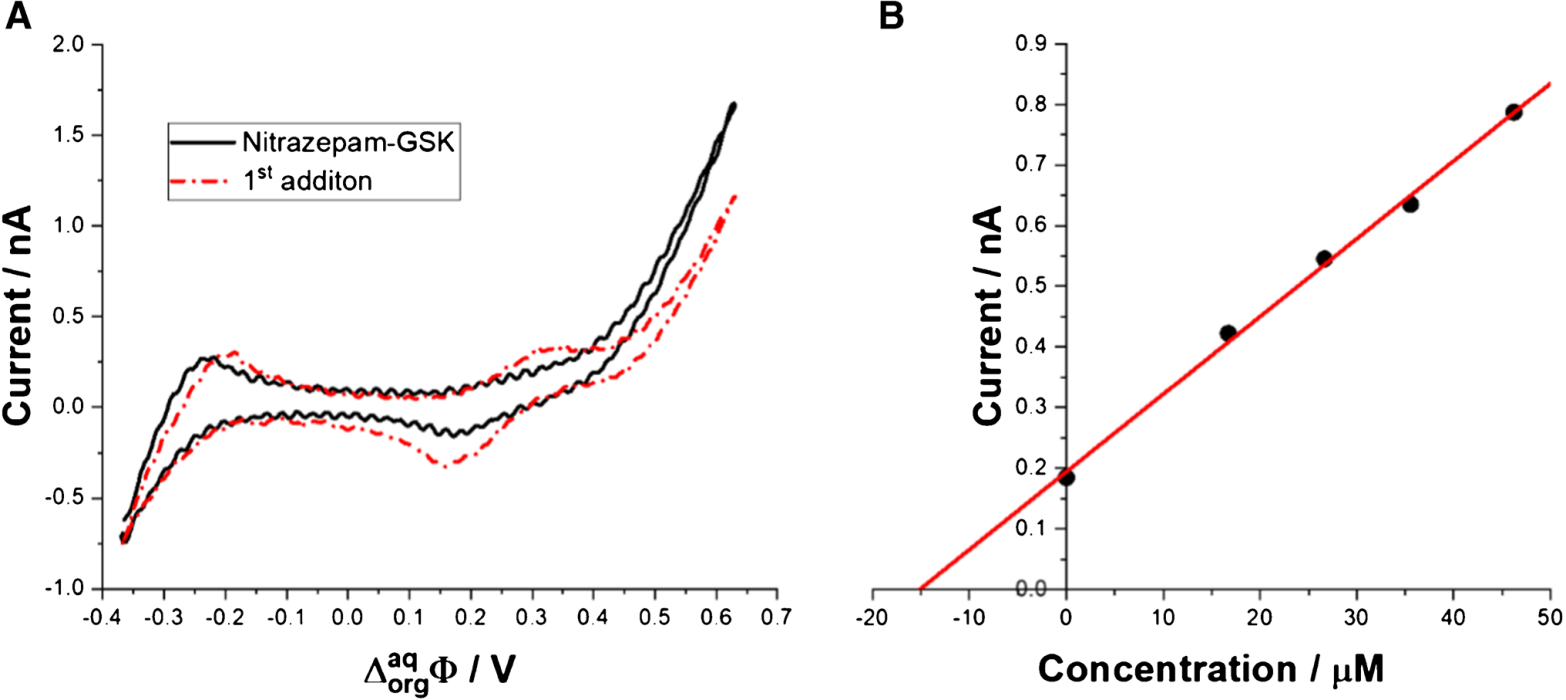
**A** ITVs recorded for the pharmaceutical formulation dissolved in 10 mM HCl and 10 mM NaCl (black, solid line) and after 1st addition of the NIT leading to a final concentration equal to 16.71 μM (red, dash-dot curve). The ITIES was miniaturized with the fused silica capillary having 25 μm as the internal pore diameter. Scan rate was set to 20 mV·s^−1^. **B** Standard addition calibration curves. Corresponding linear fit equation: *y* = 0.0128*x* + 0.1941 (*R*^2^ = 0.999)

We have used our platform to analyze the pharmaceutical formulation Nitrazepam GSK. The procedure for preparing the tablet sample was as follows. The whole pill (containing 5 mg of nitrazepam) was crushed and dissolved in 25 mL of aqueous phase. The dissolution was supported by ultrasonication for 30 min. Then, the solution was filtered using a syringe filter and diluted for measurement (20 μL of sample and 980 μL of aqueous phase). Then, in order to prepare the standard addition calibration curve, signals were recorded for samples with 4 standard additions of 2.5 μL each. The ITVs showing the blank and 1st addition, together with the standard addition calibration curve, are shown as Fig. 5A and B, respectively. After placing the sample containing the solution of the Nitrazepam GSK dissolved in 10 mM HCl and 10 mM NaCl used as the aqueous phase, we observed the signal at around 0.240 mV being fully in line with the ${\boldsymbol{\Delta }}_{\boldsymbol{org}}^{\boldsymbol{aq}}{\boldsymbol{\phi}}^{\prime }$ for NIT studied at the macroscopic ITIES. Further additions of NIT to the aqueous phase resulted in the increasing intensity of the forward and reversed current signals. Based on the obtained data, we have plotted the standard addition calibration curve which is shown in Fig. 5B. Using the liner fit equation, we have calculated the concentration of NIT in Nitrazepam GSK pharmaceutical formation that was found to be 15 μM. Based on the results of analysis and taking into account the dilution of the sample, the determined content of nitrazepam in the tested pill was 5.11 ± 0.06 mg (*n* = 3). This result is fully in line with the requirements for pharmaceutical formulation, according to which the content of the active substance should be within ± 5% of the declared value (in this case 5.00 ± 0.25 mg). The analysis showed compliance with the nitrazepam content indicated by the manufacturer further underlining utility of the proposed approach for the rapid NIT screening.

**Fig. 6:**
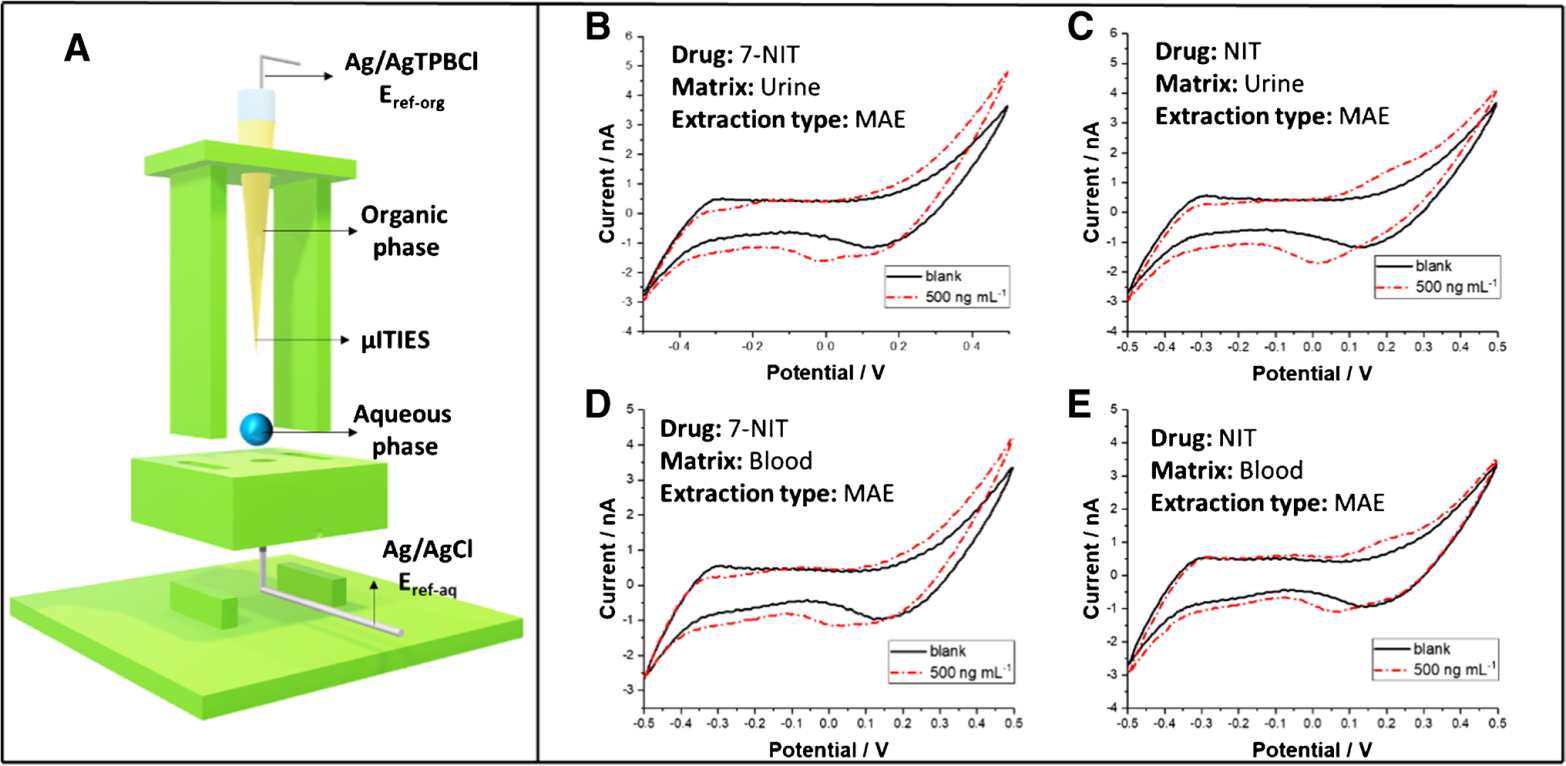
**A** 3D printed electrochemical cell used to host μITIES and dedicated for the low aqueous (up to 20 μL) and organic phase (up to 10 μL) volumes experiments. (**B**) and (**D**) are the ITVs recorded in the absence (black, solid line) and in the presence (red, dashed line) of 7a-NIT in the urine and blood extract, respectively. (**C**) and (**E**) are the ITVs recorded in the absence (black, solid line) and in the presence (red, dashed line) of NIT in the urine and blood extract, respectively. Extraction type: MAE, microwave assisted extraction. Abbreviations in part A: *E*_ref-org_, the organic phase reference and counter electrode; *E*_ref-aq_, the aqueous phase reference and counter electrode. Photography of the 3D printed cell can be found in ESI

In the last step of this work, we have applied the 3D printed cell allowing for the measurements at the liquid-liquid interface formed between the aqueous and the organic phase droplet. The benefit of the given configuration is that the cell allows for the measurements to be performed with the volumes of the reagents as little as 5 μL (for each phase). Cell construction is shown in Fig. 6A. It is composed from the micropipette tip serving as a casing of a fused silica microcapillary having the internal diameter equal to 25 μm. The tip is placed in the support with the predefined height assuring that the end of the fused silica capillary filled with the organic phase is placed in the aqueous phase droplet. The bottom compartment of the cell serves as the reservoir for the aqueous phase droplet and the Ag/AgCl wire used as the reference and counter electrode in one. Working with very small sample volumes is especially important from the biological samples point of view. Minimizing the amount of the sample to a few microliters is especially beneficial when working with body fluids excreted in small amounts (e.g., tears, sweat), but also, the samples such as blood as the analysis can be performed without the need for high quantities of blood collection. Figure 6B and D show the ITVs recorded at the drop-drop liquid-liquid interface with the aqueous phase being urine or blood samples spiked with 7a-NIT, respectively. Figure 6C and E are the analogical graphs recorded in the presence of NIT in the real sample.

We have noticed that the available potential window for the blank voltammograms (see black solid lines in Fig. 6B–E) is deprived from the prominent charge transfer reactions that could potentially interfere with the 7a-NIT and NIT detection. Nevertheless, the available potential window defined as the potential range between positive and negative limiting current has shrunk from around 0.8 V for the model systems (10 mM HCl and 10 mM NaCl used as the aqueous phase) to around 0.6 V for blood and urine. This is most probably due to the presence of a number of organic/inorganic ions at relatively high concentrations in both samples (e.g., creatine present in urine limits the available potential window on the more positive side). ITVs marked as red, dash-dot lines in Fig. 6B–E were recorded for real samples containing the 7a-NIT and NIT to real samples. Fifty μL of the reconstituted extract after extraction was applied in several portions to the 3D platform, leaving further portions for solvent evaporation. In the last portion of the extract in the volume of 10 μL, the pipette tip with the organic phase was immersed, and the measurement was carried out. Due to complexity of the real sample, possible cross reactions between analytes and the real sample matrix, and difficulty in extracting the current signals values that were attributed to the 7a-NIT and NIT transfer, we have treated our data only in qualitative manner. Table 4 shows the binary results (0, lack of a signal; 1, signal is present) for 7a-NIT and NIT added to the real samples at three different concentration: 150, 300, and 500 ng·mL^−1^. Our results indicate that we were able to detect additional portion of the ionic current for the 7a-NIT in urine for all studied concentrations, whereas for NIT, the signal was recorded only at highest concentration. This can be related with the ${\boldsymbol{\Delta }}_{\boldsymbol{org}}^{\boldsymbol{aq}}{\boldsymbol{\phi}}^{\prime }$ being higher for NIT and, hence, possibly overlying with the more positive limiting current. In the case of blood, we were able to extract additional current fraction only for the highest concentration of 7a-NIT and NIT. Our results indicate that the ITIES can be used to detect 7a-NIT and NIT is relatively simple matrix such as pharmaceutical formation. Although we were able to detect both drugs in body fluids, we needed to perform sample treatment, and detection was only qualitative. As such, we are currently working on the possible improvements combining the separation in a microscale combined with the electroanalytical detection based on the ITIES system. Finding included in this work should be useful for the overall drugs pharmacochemical evaluation and will facilitate the development of sensors focused on benzodiazepines.

**Table 4:** The results of analysis of spiked biological samples after MAE extraction obtained at the μITIES system

Biological sample	Concentration/ng·mL^−1^	Recorded signal/0/1*	
		7a-NIT	NIT
Urine	500	1	1
	300	1	0
	150	1	0
Blood	500	1	1
	300	0	0
	150	0	0

*0, lack of a signal; 1, signal is present

The tested concentration levels are higher than therapeutic concentrations expected for the blood samples which are 28–45 and 18–53 ng·mL^−1^ for NIT and 7a-NIT, respectively. As such, proposed electroanalytical procedure cannot be used to detect both analytes in blood and urine samples at therapeutic levels. However, the toxic effects in the human body are observed at blood concentrations higher than 200 ng·mL^−1^ [90]. Moreover, according to the case study [91], postmortem blood concentrations in the human body were 741 and 498 ng·mL^−1^ for NIT and 7a-NIT, respectively. Based on the same study, postmortem concentrations of NIT and 7a-NIT in urine were 498 and 1090 ng·mL^−1^, respectively. These results indicate that the developed sensor may be useful in detecting body intoxication after poisoning with nitrazepam. The proposed method selectivity is still to be fully defined. At this point, we can exclude the interference of the pharmaceutical formulation chemical constituents (lactose, starch, talc, and magnesium stearate). Also, we have found that the blood and urine samples analysis do not reveal any additional charge transfer reactions occurring within the available potential window and, hence, electrochemical interferences are absent. In our future study, we plan to examine the interfacial behavior of other membranes of the benzodiazepine drugs family.

Finally, we decided to compare the methodology proposed in this work, with the state of the art pertaining to NIT analytical determination. The result of our investigation is summarized in Table 5. The scope of analytical methods used for the nitrazepam detection covers chromatography (coupled techniques), spectroscopy, and electrochemistry. Reported limits of detection span from a decimal fraction to few microliters depending on the applied methodology. With very low operational price of the sensing procedure proposed in this work and the LOD for the nitrazepam found to be 0.4 μM (for microscopic ITIES), we offer complementary technique allowing for fast, cheap (but still rather presumptive) detection of chosen benzodiazepine drugs.

**Table 5:** Overview of the analytical methodologies used to detect nitrazepam

Method	Comment	Sample type/preparation/recovery	LOD/μM	Ref.
HPLC-UV	-	Model samples: removing the oxygen in the samples by purging with argon; recovery, N/A	0.18	[92]
HPLC-ED	-	Model samples: removing the oxygen in the samples by purging with argon was necessary; recovery, N/A	0.23	[92]
UV-VIS spectrophotometry	-	Pharmaceutical formulations: dissolution in ethanol, filtration, and dilution of the pills; recovery, 99.5–100.8%	3.30	[93]
UV-VIS spectrophotometry	-	Pharmaceutical formulations: dissolution in 4 M HCl with 1 g of zinc, filtration, and dilution of the pills; recovery, 100.0–100.9%	1.40	[94]
UV-VIS spectrophotometry	-	Pharmaceutical formulations: dissolution in ethanol, filtration, and dilution of the pills	17.80	[10]
Voltammetry	GCE-AuNPs-RGO	Serum: spiking was performed; tablet, N/A; recovery, 99.0–102.4%	0.17	[95]
This work	-	Pharmaceutical formulations: dissolution in aqueous phase, filtration, and dilution of the pills; recovery, 96.9–103.3%	2.00 (macroITIES) 0.40 (μITIES)	-

HPLC-UV, high-performance liquid chromatograph with UV-VIS detector; HPLC-ED, high-performance liquid chromatograph with electrochemical detection; AuNPs, gold nanoparticles; GCE, glassy carbon electrode; RGO, reduced graphene oxide

## Conclusions

In this work, the electrified liquid-liquid interface was used to study the interfacial behavior of two benzodiazepine drugs: 7-aminonitrazepam and nitrazepam. Both drugs were investigated in the macroscopic glass cell and with the help of a system miniaturized using fused silica microcapillaries. Ion transfer voltammetry was used as the main investigation technique. Based on the obtained electroanalytical results, we have plotted calibration curves and ion partition diagrams which allowed determination of a number of analytical and physicochemical parameters pertaining to both analytes (i.e., LODs, LOQs, voltammetric detection sensitivities, diffusion coefficients, partition coefficients, distribution constants, data defining partitioning boundary lines). The mechanism laying behind interfacial charge transfer reactions occurring in the presence of both drugs is reveled and supported with the data provided in a form of ion partition and concentration fraction diagrams. Finally, we have applied the 3D printed cell allowing for the measurements at the aqueous drop–organic drop interface (each having the volume up to a few μL) to quantitatively detect nitrazepam in the pharmaceutical formulation and further for the binary (qualitative) determination of 7-aminonitrazepam and nitrazepam in spiked blood and urine samples. Obtained results demonstrate that electrified liquid-liquid interface can be used for the determination of the both benzodiazepine drugs in studied real samples (pharmaceutical formulation, blood and urine extracts). In real-life scenarios, obtained results should be considered presumptive especially when the coexistence probability of charged drugs in the studied samples is high.

### Supporting information

Additional cyclic voltammograms and the photo of the 3D printed cell are available as the electronic supporting information. The raw data are upload to scientific repository: doi:10.5281/zenodo.7697395.

## Supplementary information

ESM 1: Additional figures.
