# Supplementary material for: Nitrazepam and 7-aminonitrazepam studied at the macroscopic and microscopic electrified liquid-liquid interface
Source: Mikrochim Acta. 2023 Apr 13;190(5):182. doi: 10.1007/s00604-023-05739-6 (PMC10101902; doi:10.1007/s00604-023-05739-6)
Supplement: Supplementary file 1 — Additional figures. [file 604_2023_5739_MOESM1_ESM.docx]

**Electronic Supplementary Material**

**Nitrazepam and 7-aminonitrazepam studied at the macroscopic and microscopic electrified liquid-liquid interface**

Paweł Stelmaszczyk,^a^ Karolina Kwaczyński,^b^ Konrad Rudnicki,^b^ Sławomira Skrzypek,^b^ Renata Wietecha-Posłuszny,^a^* Lukasz Poltorak ^b^*

1. Laboratory for Forensic Chemistry, Department of Analytical Chemistry, Faculty of Chemistry, Jagiellonian University, Gronostajowa 2, 30-387 Krakow, Poland
2. Electrochemistry@Soft Interfaces Team, Department of Inorganic and Analytical Chemistry, Faculty of Chemistry, University of Lodz, Tamka 12, 91-403 Lodz, Poland

***First corresponding author:** lukasz.poltorak@chemia.uni.lodz.pl

***Second corresponding author:** renata.wietecha-posluszny@uj.edu.pl

**Key words:** date rape drugs; psychoactive chemicals; voltammetry; ITIES; sensor

**Table of contents:**

1. Scan rate dependency for the 7a-NIT and NIT. – page 2
2. ITVs recorded for 7a-NIT and NIT at different aqueous phase pH vales. – page 3
3. SEM image of the fused silica capillary and the photo of the 3D printed cell. – page 4


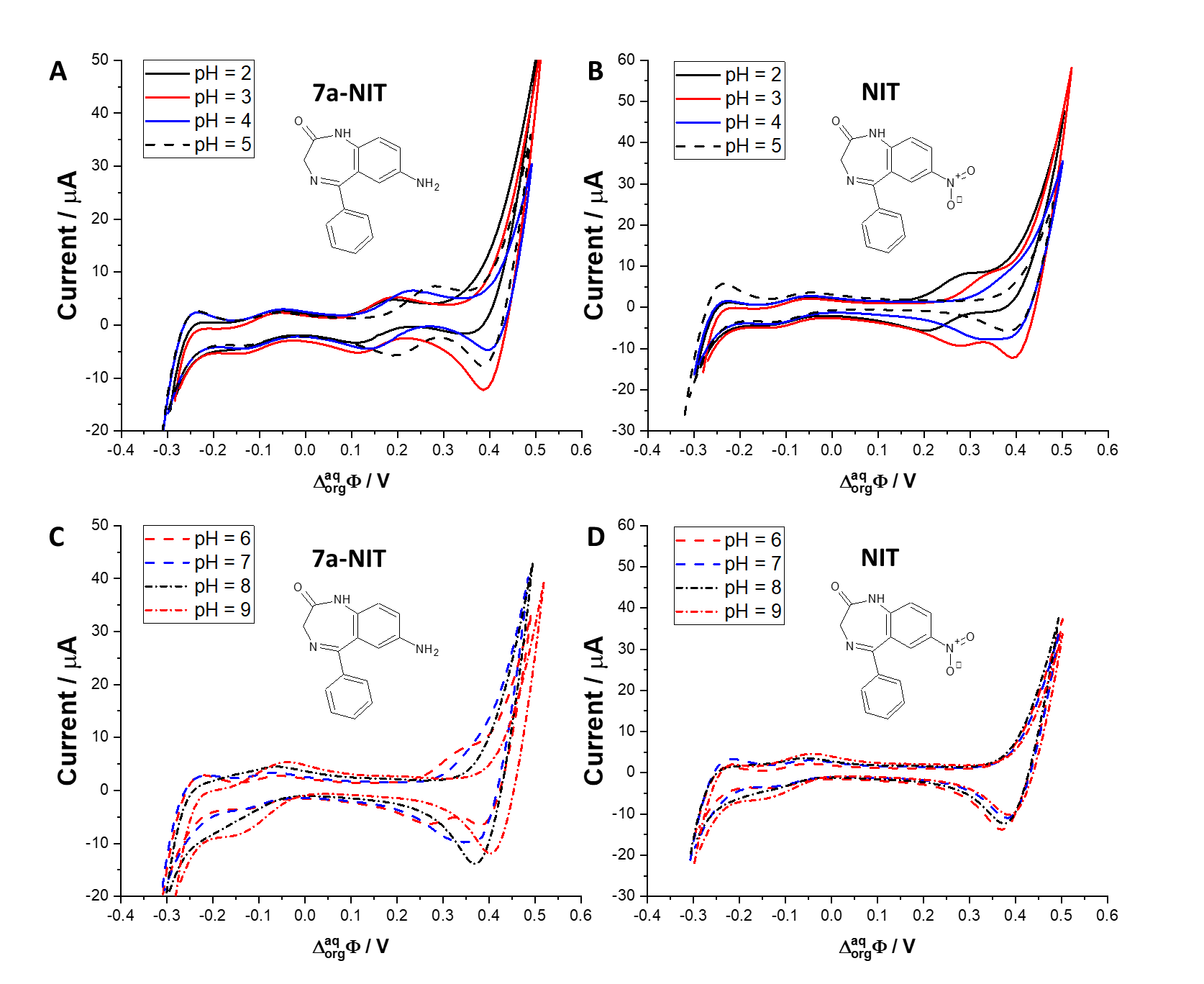


**Figure S1.** Ion transfer voltammograms recorded for 7a-NIT (A, C) and NIT (B, D) [benzodiazepine] = 30 µM, at different aqueous phase pH values (pH value indicated in the legend; the aqueous phase was the 10 mM NaCl BRB solution). Scan rate: 20 mV·s^-1^. All curves were recorded in the presence of the 40 µM TPrACl added to the aqueous phase as the internal reference aiming at potential axis calibration to the formal Galvani potential difference scale.


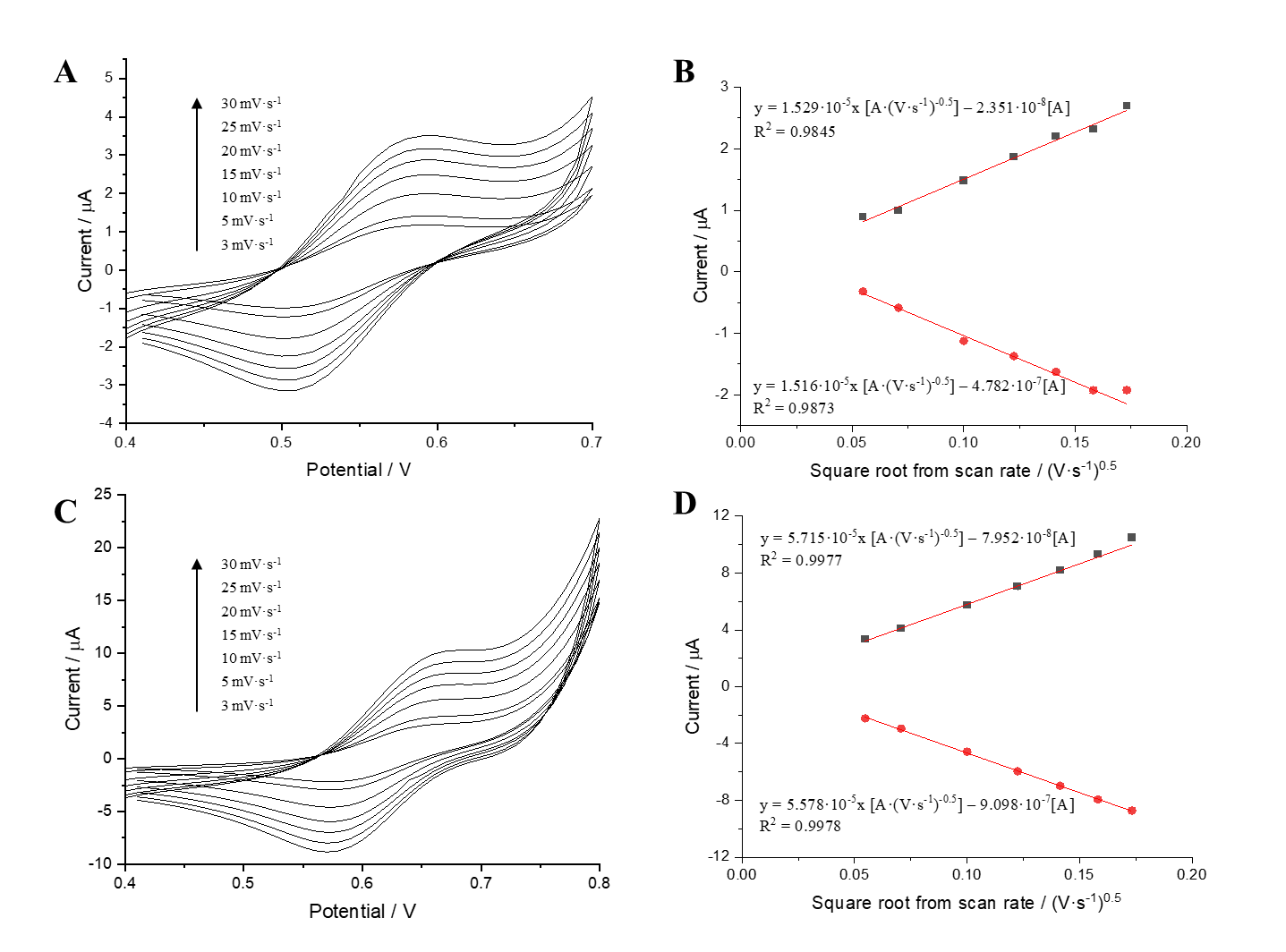


**Figure S2.** A and C are the ion transfer voltammograms recorded for the fixed concentration of 7a-NIT and NIT; [benzodiazepine] = 30 µM; and increasing scan rate in the range from 3 to 30 mV·s^-1^ (the order of applied potential scan rates corresponds to the sequence of depicted curves). B (7a-NIT) and D (NIT) are the positive and negative current signals plotted in function of the square root of the applied potential scan rate. Liner fit equation are placed next to the corresponding data set.


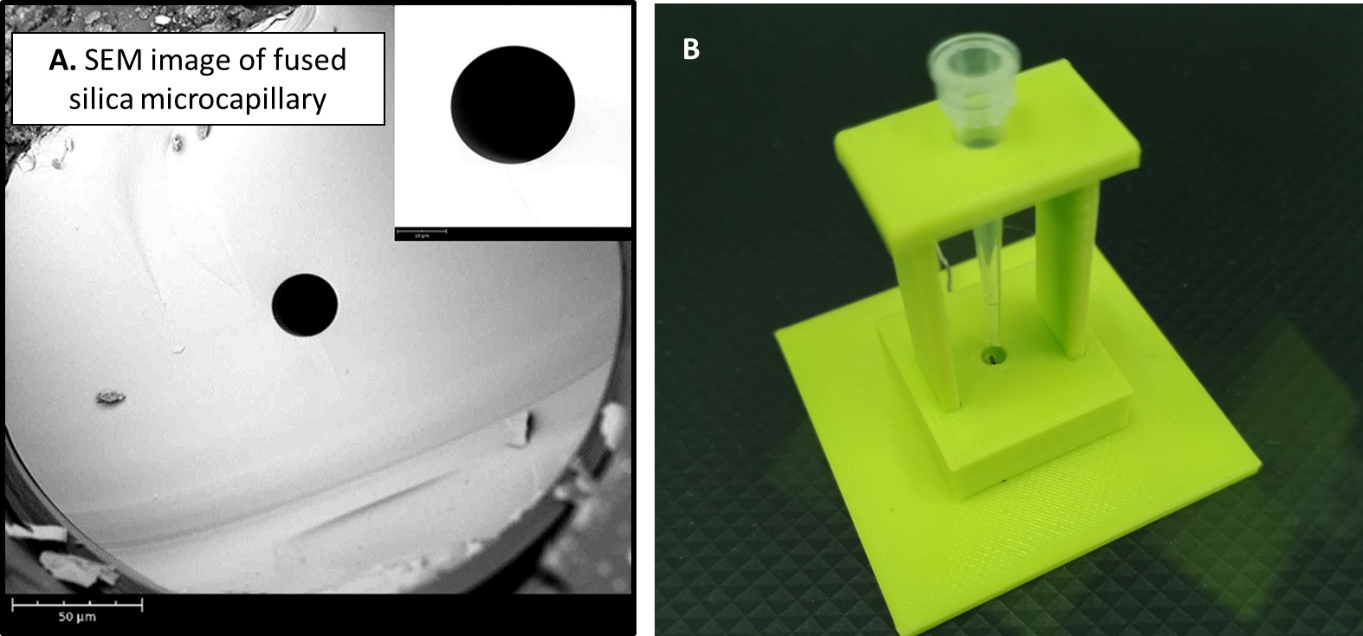


**Figure S3.** A – SEM image of the fused silica microcapillary inserted into micropipette tip. Scale bare in the inset image is 10 µm. B – Photo of the 3D printed cell.
